# *Aloe djiboutiensis*: Antioxidant Activity, Molecular Networking-Based Approach and In Vivo Toxicity of This Endemic Species in Djibouti

**DOI:** 10.3390/molecules26103046

**Published:** 2021-05-20

**Authors:** Abdirahman Elmi, Fatouma Mohamed Abdoul-Latif, Rosella Spina, François Dupire, Stéphanie Philippot, Champy Marie-France, Hugues Jacobs, Dominique Laurain-Mattar

**Affiliations:** 1 Université de Lorraine, CNRS, L2CM, 54000 Nancy, France; abelfourreh@hotmail.com (A.E.); rosella.spina@univ-lorraine.fr (R.S.); francois.dupire@univ-lorraine.fr (F.D.); stephanie.philippot@univ-lorraine.fr (S.P.); 2 Centre d’Etudes et de Recherche de Djibouti, Medicinal Research Institute, IRM-CERD, Route de l’Aéroport, Haramous B.P. 486, Djibouti City, Djibouti; fatouma_abdoulatif@yahoo.fr; 3 PHENOMIN-ICS, Institut Clinique de la Souris, Université de Strasbourg, 67404 Illkirch, France; champy@igbmc.fr (C.M.-F.); hugues@igbmc.fr (H.J.)

**Keywords:** *Aloe djiboutiensis*, anthraquinones, polyphenols, antioxidant, molecular networking, mass spectrometry, histopathology, serum enzymes, cytotoxicity

## Abstract

For the first time, the study of the antioxidant activity, the characterization of the phytoconstituants, and the evaluation of in vitro and in vivo toxicity of *A. djiboutiensis* leave and latex are performed. The antioxidant activity of both latex (ADL) and the methanolic extract of leaves (ADM) is determined using 1-diphenyl-2-picrylhydrazyl (DPPH), 2,2′-azino-bis 3-ethylbenzothiazoline-6-sulphonic acid (ABTS) scavenging radical methods and ferric reducing/antioxidant power (FRAP) assay. The phytochemical study of latex is done using Liquid Chromatography-Mass Spectrometry (LC-MS/MS) and a molecular networking-based approach. The evaluation of in vivo toxicity is performed on mice by oral gavage with a suspension of ADL. Our results show that weak antioxidant activity of ADL and ADM in opposition to their high polyphenol, 83.01 mg and 46.4 mg expressed in gallic acid equivalent (GAE)/g of dry weight (DW), respectively, and flavonoid contents 13.12 mg and 4.25 mg expressed in quercetin equivalent (QE)/g dry weight (DW), respectively. Using the Global Natural Products Social Molecular Networking (GNPS) website, nine (9) anthraquinones derivatives, ten (10) chromones derivatives, two (2) flavonols/ chromones isomers are annotated in the molecular network. The treated mice do not display abnormalities in their general physical appearance and biochemistry parameters, compared to the controls. Only glucose and calcium levels are slightly higher in male treated mice compared to the vehicles.

## 1. Introduction

**Figure 1:**
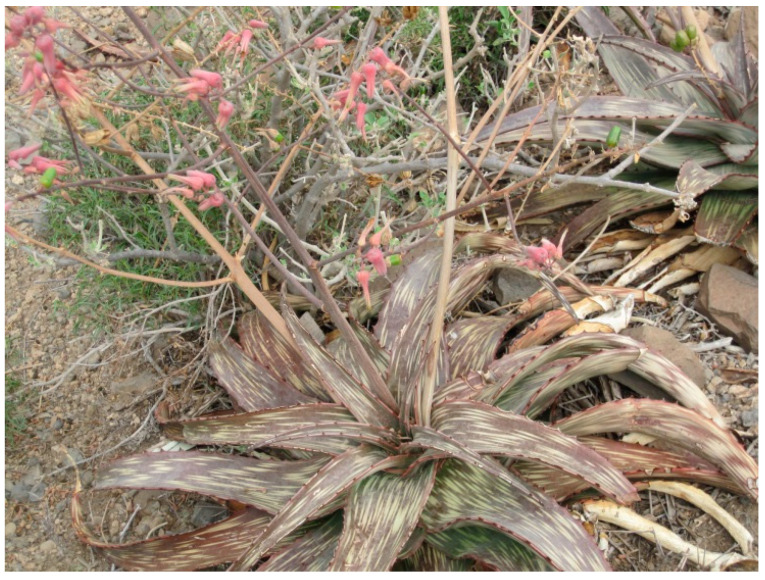
*Aloe djiboutiensis* plant.

The Aloe genus, belonging to the Xanthorrhoeaceae family, is composed of approximately 140 perennial succulent species found mostly in arid places as in Africa and India [1]. *Aloe vera* is the widely used species; however, different species are used in traditional medicine like *A. arborescens*, *A. perryi*, *A. andongensis*, and *A. ferox* [2]. *Aloe vera* is traditionally used in South Africa for skin disorders, constipation, and wounds [3,4,5]. Aloe plants are used for their different biological activities, which render them very important from an economic point of view [1]. These plants are known to biosynthesize polysaccharides components [6,7], and specialized metabolites with interesting biological activity as mainly phenolic compounds, including chromones, anthraquinones, and pyrones. These compounds have multidiscipline pharmacological applications, such as antitumor, antidiabetic, antityrosinase, and antiulcer activities [8,9,10]. The cinnamoyl-*C*-glucosyl-chromone, isolated from *Aloe barbadensis* showed an antiinflammatory effect [11]. However, some compounds, like anthraquinones, can be toxic in living organisms [8,12,13]. All these compounds are located in different parts of the leaves, which are heterogeneous, namely: The outer green epidermis, the pulp below the epidermis containing the bitter latex or sap, and the inner leaf pulp consisting of aloe gel [8]. Anthraquinones are present in the three parts of the leaves, while acemannan polysaccharides are concentrated in aloe gel, as well as alkaloids and polyphenols. Phenolic compounds were extracted from the latex [8]. *Aloe djiboutiensis* is a native plant in Djibouti (Figure 1), named botanically recently in 2007 [14]. This plant is located in the mountainous regions of Djibouti with an arid climate, especially in the localities of Arta and Day, in the center and north of the country, respectively, and was also found in the border country, Eritrea. *Aloe djiboutiensis* can reach heights of up to 61–99 cm. It is a succulent plant with a basal rosette leaf, speckled with elongated whitish spots. The latex is orange-yellow. The flowers are pink before turning yellow, and the flowering duration may vary from May to June [15].

*Aloe djiboutiensis* is widely used in traditional medicine for a long time locally against eye infections and as a laxative. However, phytochemical studies and biological evaluation of this plant are non-existent. Considering the widespread use of *Aloe djiboutiensis* in Djibouti, the present research work was conducted to assess the possible toxic effect and antioxidant activity of the latex and the leave extract, as well as to detect rapidly the previously characterized known molecules in the genus *Aloe* and to create a metabolic fingerprinting of *A. djiboutiensis*. The phytochemical profile of *A. djiboutiensis* latex was evaluated using LC-MS/MS analysis. The data obtained from tandem mass spectrometry were analyzed using the Global Natural Product Social Molecular Networking (GNPS) website [16]. GNPS is an interactive platform to work using untargeted mass spectrometry data, to process the data, to attributing a possible chemical structure to a detected small molecule in the complex matrix, such as plant extracts. The platform permitted to connect the MS/MS fragmentation and to create Molecular Networking.

## 2. Results and Discussion

### 2.1. Antioxidant Activity

The latex and the methanolic extract of *A. djiboutiensis* leaves were investigated to determine their in vitro antioxidant activity using the DPPH, ABTS, and FRAP assays, and results are presented in Table 1. The latex and the methanolic extract showed no or weak antioxidant activity in DPPH (inhibition concentration at 50%, IC_50_ < 1000 µg/mL) and ABTS essays (IC_50_ = 600 ± 1.8 and 632 ± 3.2 µg/mL, respectively). Moreover, both samples showed weak activity considering FRAP assay (1.50 ± 0.12 and 1.68 ± 0.20 mg equivalent vitamin C (EVC)/g DW, respectively).

These results obtained with the newly discovered *Aloe* species are in opposition to the works carried out on extracts from leaves of other species of *Aloe*. It has been reported that *A. ferox* [17], *A. pillansii*, *A. broomii*, *A. spinosissima*, and *A. arborescens* [18] leaf extracts exhibited high antioxidant activity. Moreover, different parts of the *A. vera* plant, like leaf epidermis, flowers, and gel, showed in vitro antioxidant effects [19,20]. The differences in the antioxidant activity in aloe also depends on numerous factors, such as the type and conditions of cultivation, harvest time, climate, the position of leaves on the stem, aloe species, and the method used for harvesting leaves [5,21].

The weak antioxidant activity measured in this in vitro study could be explained by the chemical constituents in the latex and the methanolic extract of *A. djiboutiensis* leaves. It has been reported that the antioxidant activity is different depending on the anthraquinones [22]. Indeed, anthrone showed a strong antioxidant activity (98% inhibition of peroxidation), while emodin showed low activity (36%), and chrysophanol even had a prooxidant action (−23%).

### 2.2. Total Polyphenol and Total Flavonoid Contents

The total polyphenol contents of latex and leave methanolic extract of *A. djiboutiensis* were determined (Table 1). The highest amount was detected in the latex with 83.01 mg GAE/g DW), while the methanolic extract showed the amount of 46.4 mg EAG/g DW. Results of total flavonoid contents in both samples also revealed that the latex has the highest level of flavonoid content (13.12 mg QE/g DW), the leaves extract displayed three less total flavonoid content (4.25 mg QE/g DW) than in latex (Table 1). Comparing these results with previous studies, *A. djiboutiensis* leaves extract contain higher polyphenol content than *A. vera* leaves extract (3.07 mg/g DW) [23]. It is known that there is a relation between the content of phenolic compounds and the antioxidant properties, as shown in *A. vera* leaf skin extract [19]. However, despite very high polyphenol content levels in latex and leaves of *A. djiboutiensis*, the antioxidant activity is low.

### 2.3. Identification of Specialized Metabolites Using LC-MS/MS Molecular Networking Based-Approach

**Figure 2:**
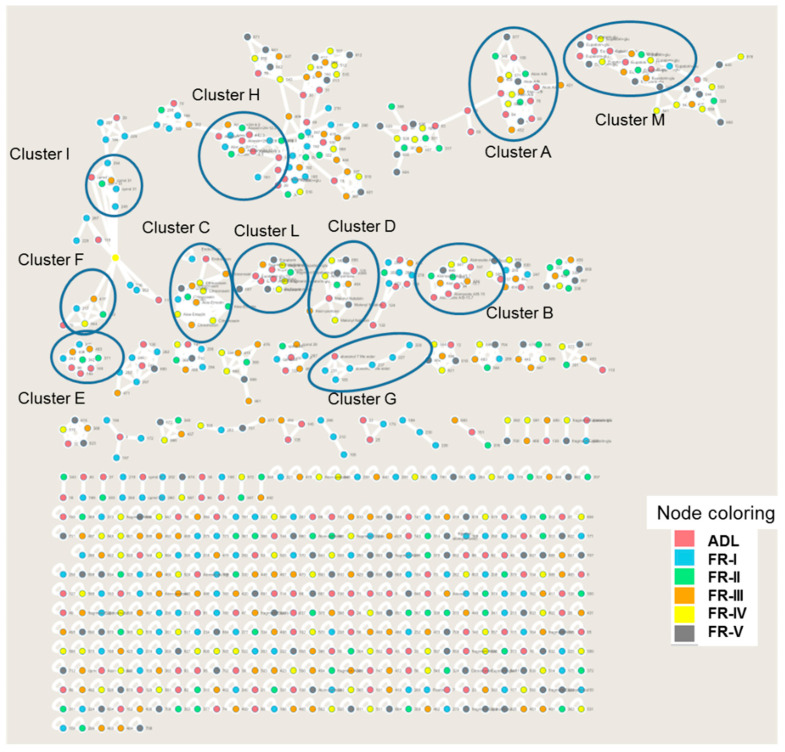
Global molecular network of ions produced after MS/MS analysis of latex (ADL) and five purified fractions. The network is realized using the GNPS website and visualized using Cytoscape. Each sample is represented by one color. The nodes of ADL, the latex of *A. djiboutiensis,* are annotated in red; the nodes of the fraction FR-I are annotated in clear blue; the nodes of the fraction FR-II are annotated in green; the nodes of the fraction FR-III are annotated in orange; the nodes of the fraction FR-IV are annotated in yellow; the nodes of the fraction FR-V are annotated in grey. Eleven (11) annotated clusters (from A to M) are highlighted by a blue circle. Other clusters not annotated correspond to non-identified compounds.

The latex (ADL) and five fractions (from FR-I to FR-V), obtained after LH-20 fractionation of latex, were analyzed, and the phytochemical profile is evaluated using LC-MS/MS analysis. The identification/annotation of the chemical compounds is based on the comparison of commercial standards, the chemicals present in our in-house database (DataNat database, n° IDDN: FR.001.480019.000.S.P.2020.000.10300) and GNPS (Global Natural Products Social Molecular Networking). GNPS is used for the generation of molecular networking, and Cytoscape software 3.8.0 (U.S. National Institute of General Medical Sciences, Bethesda, MD, USA) [24] is used for the visualization of them. Molecular networks organize and visualize MS/MS data based on spectral similarity based on the presence of homologous MS/MS fragments or homologous neutral loss. In total, 712 nodes are visible in molecular networking, which included 67 clusters (node ≥ 2). The observation of molecular network reveals the presence of eleven (11) annotated clusters named, respectively A, B, C, D, E, F, G, H, I, L, and M. The comparison to GNPS spectral databases allowed annotating has according to the chemical structures: Anthraquinones (in clusters A, B, C, and D), chromones (in clusters E, F, G, H, and I), flavonols/chromones isomers (in cluster L and M). All the clusters are visible in Figure 2.

All results from the molecular networking-based approach using LC-MS/MS analysis are presented in Table 2.

**Figure 3:**
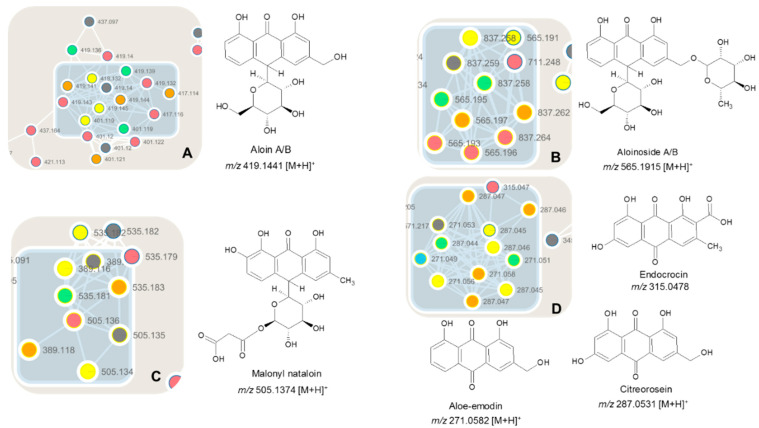
Antraquinones clusters, cluster A, cluster B, cluster C, cluster D in latex of *A. djiboutiensis*.

GNPS efficiently separated anthraquinones from each other, such as aloin A/B (cluster A), aloinoside A/B (cluster B), malonyl nataloin and aloin pentose (cluster C), and aglycone of anthraquinones (cluster D) as illustrated in Figure 3.

In cluster A, the presence of monoglucosilate antraquinones are observed, especially aloin A/B with [M + H]^+^ of 419.1441 *m/z* were annotated. Aloin A/B (with retention times of 14.7 min and 15.1 min) are known compounds in *A. djiboutiensis* latex (ADL), and they are present in the fractions from FR-II to FR-V. The characteristic MS/MS fragmentation is *m/z* 257.0796 [M-glycosyl + H]^+^, 239.0768 [M-glycosyl-H_2_O + H]^+^, 211.0785 [M-glycosyl-H_2_O-CO + H]^+^ (Figure S1). The presence of aloin A/B, as compounds **1A** and **1B**, is also confirmed using the original standard present in our database DataNat and by Nuclear Magnetic Resonance (NMR) spectroscopy of the fraction FR-II (Figures S2 and S3).

In cluster B, diglucosilate antraquinones are observed, and aloinoside A/B (*m/z* 565.1915 [M + H]^+^) were annotated. These compounds (**2A** and **2B**) are the *O*-rhamnoside derivatives of aloin A/B. Clearly, in the spectra, the loss of the neutral rhamnosyl unit (146.0579 Da) followed by the loss of neutral glycosyl unit (162.0528 Da) are observed.

In cluster C, compound **3**, with *m/z* 505.1374 [M + H]^+^, was identified as malonyl nataloin, a uncommon anthraquinone in the *Aloe* genus [25]. The MS/MS fragmentation shown the neutral losses of 180.0634 Da and 86.0004 Da indicated the presence of glycosyl unit and malonyl-H_2_O unit. Compound **4** with *m/z* 389.1213 was tentatively identified as aloin-pentose, following the loss of pentosyl-H_2_O.

It should be noted that aloin A/B, aloinoside A/B, and malonyl nataloin belong to the family of C-glycosyl compounds.

In cluster D, compounds **5**, **6**, and **7** with *m/z* 315.0478, 271.0582, and 287.0531 were tentatively identified as three anthraquinones aglycons, such as endocrocin, aloemodin, and citreorosein, respectively. The difference between aloemodin and citreorosein is hydroxyl (OH), which corresponds to the molecular formula of C_10_H_10_O_5_ and C_10_H_10_O_6_, respectively. They are present principally in the fractions FR-III and FR-IV. The identity of aloemodin is also confirmed using the original standard present in our database, DataNat.

Compounds found in clusters E to I are series of isoaloesin and aloesinol derivatives.

The molecular networking-based approach reveals that the latex of *A. djiboutiensis* (ADL) is rich in chromones C-glucoside derivatives.

**Figure 4:**
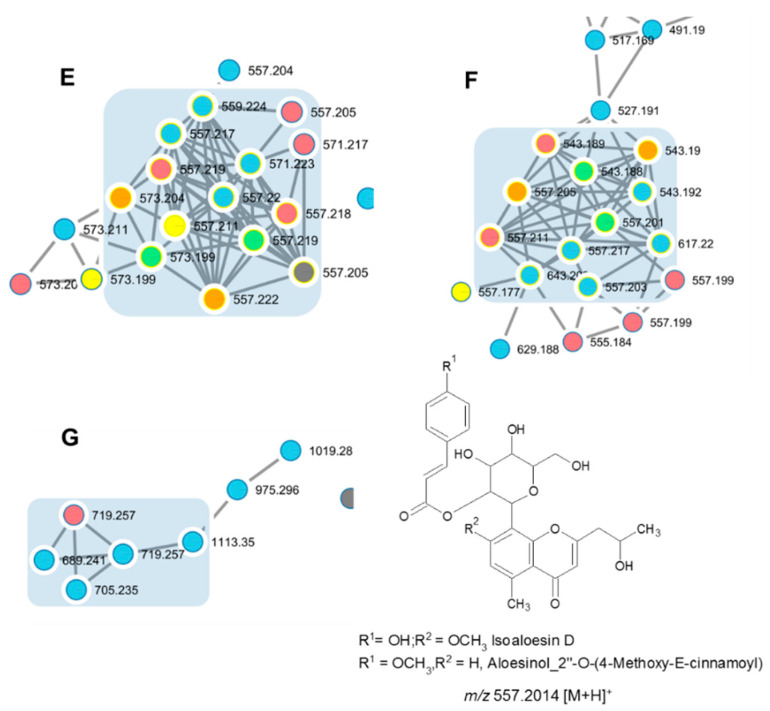
Clusters E, F, and G of chromones derivatives.

Compounds **8**, **9**, and **10**, present in clusters E, F, and G, are concentrated in the fraction FR-I, the node is visualized with blue color, connected with latex sample ADL, visualized with red color (Figure 4).

Compounds **8** and **9A**/**9B** have the same molecular formula [C_29_H_32_O_11_ + H]^+^ with *m/z* 557.2014, but different retention times and MS/MS fragmentation.

In cluster E, the identification proves the presence of compound **8** or isoaloeresin D. The fragmentation pattern is 513.1783 [M-CH_2_CHOH + H]^+^, 349.1291 [M-CH_2_CHOH-coumaric + H]^+^, 217.0876 [M-CH_2_CHOH-coumaric-pentosyl + H]^+^, 187.0727 [M-CH_2_CHOH-coumaric-glycosyl + H]^+^. Moreover, in the MS/MS spectrum, it is possible to observe the fragment at 437.1589 [M-part of sugar + H]^+^, 393.1374 [M-part of sugar-CH_2_CO + H]^+^ and 247.0963 [M-part of sugar-CH_2_CO-(coumaric-H_2_O) + H]^+^. The loss of 120.0423 Da fragment is characteristic of chromones C-glucoside, according to MS/MS fragmentation ions induced by the cleavage of sugar unit, present in position 7 (Figure S4 in Supplementary Materials).

In addition, the fraction FR-I is analyzed by NMR. In the proton and the carbon NMR, the characteristic signals of isoaloeresin D are observed (Figures S5 and S6).

In the same cluster, compound **14** or Aloesinol_7-Me_ether, 2″-*O*-(3,4-dihydroxy-*E*-cinnamoyl) is identified with *m/z* 573.1982 [M + H]^+^. The fragment at *m/z* 163.0378 is characteristic of loss of caffeic group or 3,4-dihydroxycinnamic moiety [caffeic-H_2_O + H]^+^.

In cluster F, the MS/MS spectrum of compound **9A** and **9B** or aloesinol_2″-*O*-(4-methoxy-cinnamoyl) derivatives, show the loss of a fragment of 44.0262 Da (CH_2_CHOH), followed by the loss of methoxycinnamate (178.0630 Da) (Figure S7). In other hand it possible to observe the fragments at 513.1783 [M-CH_2_CHOH + H]^+^, 335.1101 [M-CH_2_CHOH-MeO-cinnamoyl + H]^+^ and 203.0672 [M-CH_2_CHOH-MeO-cinnamoyl-pentosyl + H]^+^. The *m/z* 161.059 indicates the 4-methoxybenzene or 4-methoxy-cinnamoyl fragment.

In cluster G, compound **10A** and **10B**, called isoaloeresin d glycosyle derivatives, showed the presence of coumaroyl group, and a difference of neutral fragment of 162.0528 Da between *m/z* 719.2570 [M + H]^+^ and *m/z* 557.2033 [M-glycosyl + H]^+^ (Figure S8). This indicates that compound **10** is a derivative of compound **8** (isoaloeresin D). In both cases, an MS/MS spectrum *m/z* 147.0427 [coumaric acid-H_2_O + H]^+^ corresponds to the loss of a coumaric fragment.

In cluster H, the presence of compound **12** or aloesin (*m/z* 395.1339 [M + H]^+^) and compound **13**, a derivative with one glycoside unit more in comparison to aloesin (*m/z* 557.1874 [M + H]^+^), are observed.

In cluster I, compound **15** has *m/z* 543.1908 [M + H]^+^. It is identified as aloesinol_2″-*O*-(4-Hydroxy-*E*-cinnamoyl). The fragment *m/z* 147.0427 [coumaric acid-H_2_O + H]^+^ corresponds to loss of coumaric moiety.

The compounds identified using DEREPLICATOR+, in the GNPS website, are present in a recently published review [26]. Using the chemical structures isolated from the Genus Aloes and the in silico study reveals that several compounds are potential actives against the Severe Acute Respiratory Syndrome coronavirus (SARS-CoV-2). It is noted that in ADL, eight (8) compounds are detected, such as aloinoside A/B (compounds **2A/2B**), aloesinol_2″-*O*-(4-methoxy-cinnamoyl) derivatives (compounds **9A/9B**), isoaloeresin d glycosyle derivatives (compounds **10A/10B**), aloesinol_7-Me_ether,_2″-*O*-(3,4-dihydroxy-*E*-cinnamoyl) (compound **14**), and aloesinol_2″-*O*-(4-Hydroxy-*E*-cinnamoyl) (compound **15**).

In clusters L and M, flavonols/chromones isomers are detected, in particular, an aglycon and its heteroside glycosylated: Compound **16** with *m/z* 345.0953 [M + H]^+^ and compound **17** with *m/z* 507.1505 [M + H]^+^. The putative identification is oriented for eupatorin or aglycon of pendulin (compound **16**) and eupatorin-glycosyle or pendulin (compound **17**).

**Figure 5:**
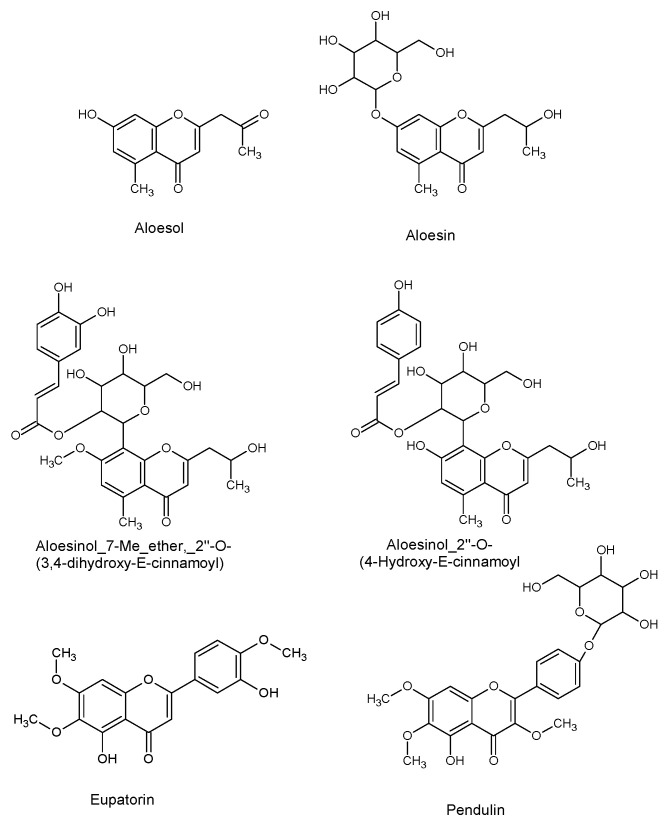
Chemical structures of aloesol (compound **11**), aloesin (compound **12**), Aloesinol_7-Me_ether,_2″-*O*-(3,4-dihydroxy-*E*-cinnamoyl) (compound **14**), Aloesinol_2″-*O*-(4-Hydroxy-*E*-cinnamoyl) (compound **15**), eupatorin, and pendulin (compounds **16/17**).

Latex sample (ADL). Cmpds is acronyme for compounds. All compounds are identified by GNPS, except for compound **3** (Malonyl nataloin), which was identified manually according to MS/MS fragmentation. DT, the identification is performed using commercial standards and in house database DataNat. D+: Using DEREPLICATOR +. M: Manually tentative identification by MS/MS fragmentation. It is known that, for radical scavenging activity, the carbonyl group of chromone and the two dihydroxy groups present in catechol (ring B, position 3’ and 4’) along with hydroxyl groups in C-3 and C-5 position are important. When methylation/glycosylation of the hydroxyl groups on the chromone nucleus is present, the radical scavenging potential decreases notably [27]. In our case, several chemical structures (Figure 5) have a high level of methylation, which can explain the low antioxidant activity calculated from the latex sample (ADL). In addition, aloin A/B (compound **1A** and **1B**) and aloe-emodin (compound **6**) were detected in the latex. It has been reported that aloe-emodin is an antioxidant at some concentrations and became prooxidant at other concentrations, as described by Tian and Hua [28].

In the literature, the compounds present in *Aloe vera* crude extract can be implicated in antioxidant or prooxidant activity. The capacity of aloe-emodin and aloin to be antioxidant or as a prooxidant depends on their concentrations. Some conditions are known to favor prooxidant activity, such as high aloe emodin, low aloin, high cinnamic acid, and low anthrone contents [29].

In conclusion, 21 compounds are putatively identified by molecular networking-based approach and GNPS libraries. Aloin A/B and aloemodin are confirmed using the DataNat database. For aloin A/B and isoaloeresin D, the characteristic signals are visible in ^1^H and ^13^C-NMR.

### 2.4. In Vitro Toxicity

Possible toxicity of latex (ADL) and leaves methanolic extract (ADM) of *A. djiboutiensis* at the concentration of 256 μg/mL was evaluated against embryonic lung MRC-5 cell line using MTT test. Latex did not show any toxicity against MRC-5 cells with 99 ± 4.7% of the surviving cells, while leaves methanolic extract showed low toxicity at this concentration of 256 μg/mL (88.25 ± 4.95% of cell survival). However, latex showed the highest contents of both total polyphenolic compounds and flavonoids comparatively to the leaves methanolic extract. These results suggest that polyphenols are not responsible for the cellular toxicity, and the moderate cytotoxicity observed in leaves may have been contributed by other metabolites in the methanolic extract. *Aloe vera* latex and leaves contain multiple constituents with potential toxicological activities, such as anthraquinones, which showed cytotoxicity [2].

### 2.5. In Vivo Observations

A search for possible in vivo toxicity of latex of *A. djiboutiensis* was performed on mice.

**Figure 6:**
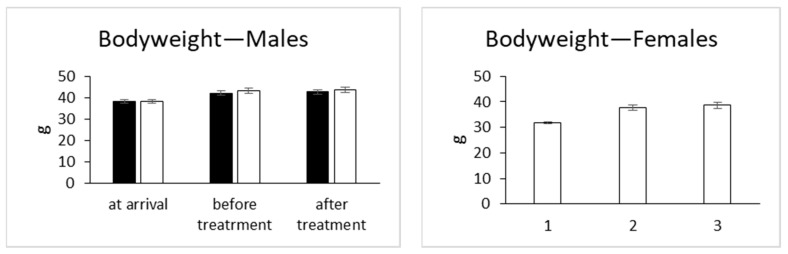
Bodyweight of mice given (ADL) latex of *A. djiboutiensis*. Data are mean ± standard error of *mean* (SEM) of 20 mice (10 males and 10 females) for treated mice and 20 mice (10 males and 10 females) for non-treated mice. The data from treated mice were compared to the vehicle mice using an unpaired Student’s *t*-test.

Bodyweight was recorded upon arrival (10 weeks of age), just before treatment (16 weeks of age), and 1 week after treatment. Bodyweight was not significantly changed between treated and vehicle mice in both sexes (Figure 6). Bodyweight changes are indicators of adverse side effects, and especially a loss of more than 10% of the initial bodyweight is a bad sign [30].

The treated mice did not display morphological abnormalities in their general physical appearance and body shape compared to the controls (weight, length, and obvious dysmorphology in the physical appearance, i.e., tail kinks, shape of ears, eyes, head, teethes, limbs, number and shape of digit, irregularities and variation in coat color, hair distribution and development, irregularities in the genitals).

**Figure 7:**
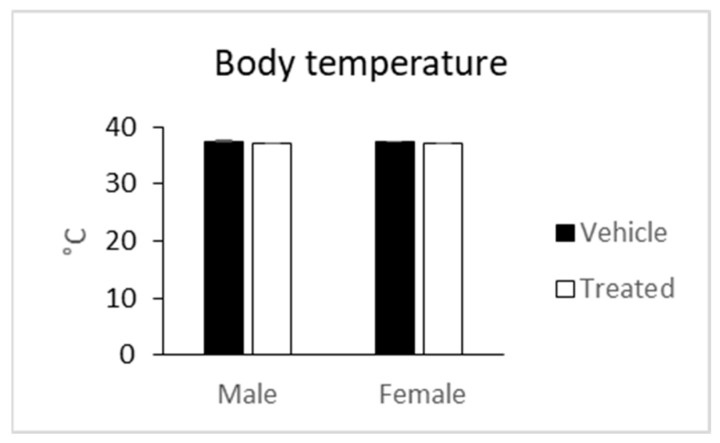
Body temperature of mice given latex of *A. djiboutiensis*. Data are mean ± standard error of *mean* (SEM) of 20 mice (10 males and 10 females) for treated mice and 20 mice (10 males and 10 females) for non-treated mice. The data from treated mice were compared to the vehicle mice using an unpaired Student’s *t*-test.

Body temperature was not changed too in treated mice compared to the vehicle mice in both sexes (Figure 7). Some signs of toxicity, such as decreased locomotion, intermittent diarrhea, loss of appetite, were observed in mice fed with latex (5 g/kg) of *A. pulcherrima* Gilbert and Sebsebe after two weeks of treatment [31].

### 2.6. Biochemistry Parameters

**Figure 8:**
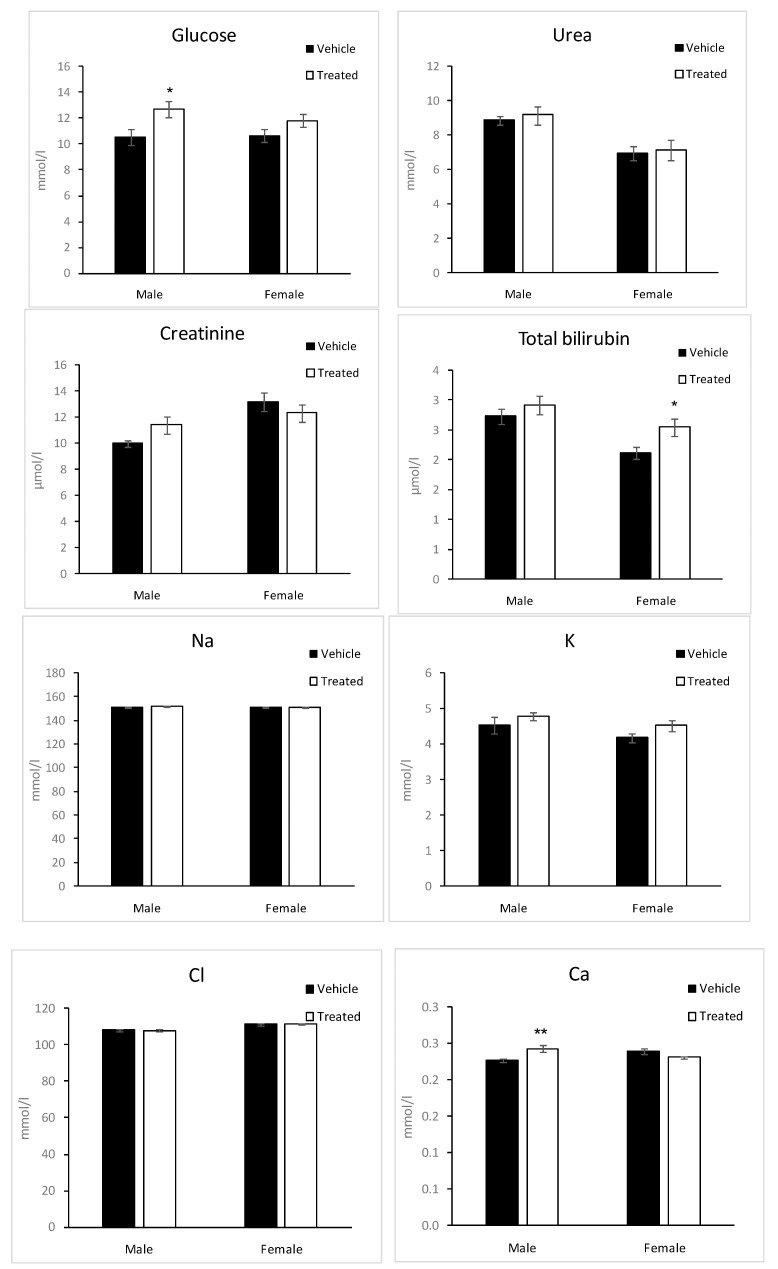

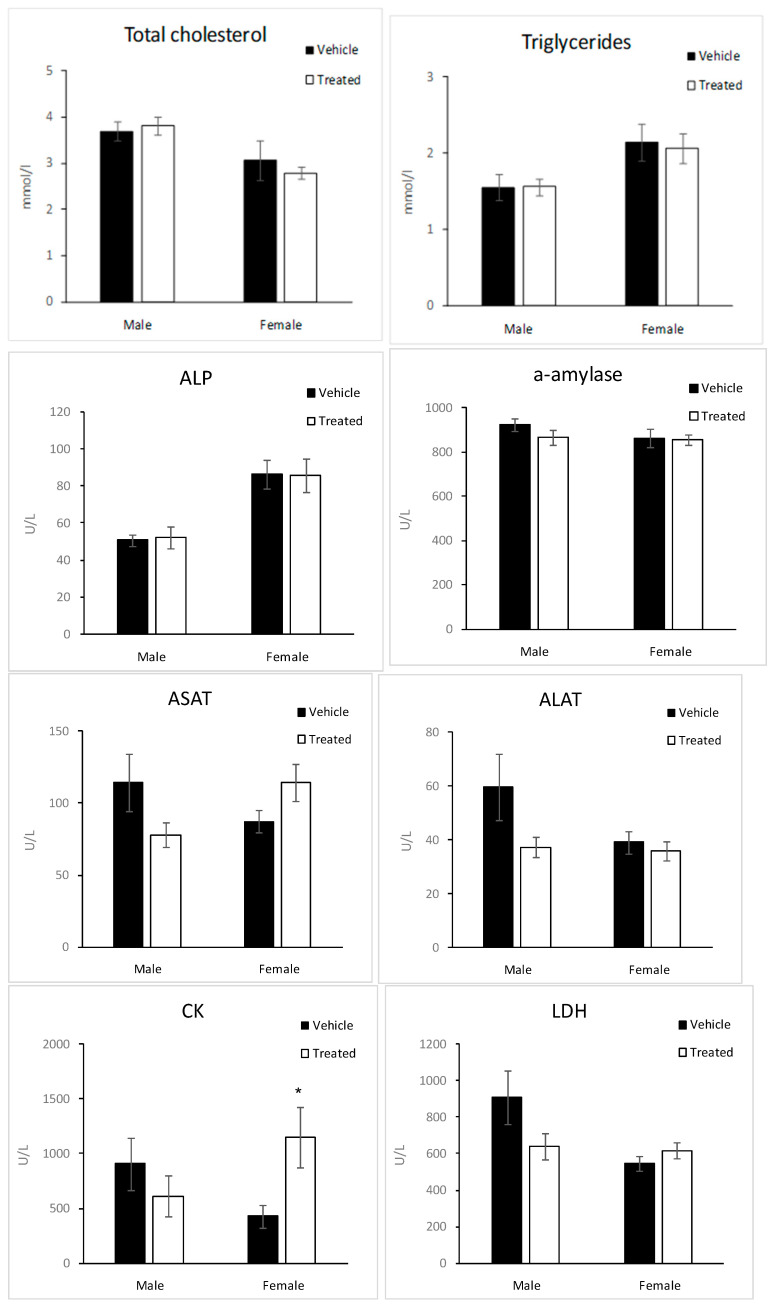

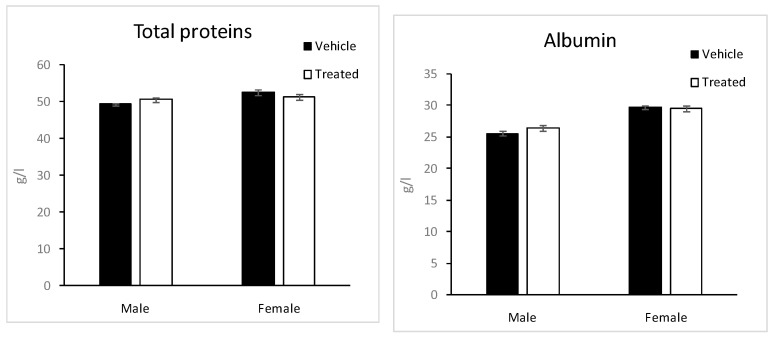
Biochemical changes in plasma of mice given latex of *A. djiboutiensis*. Data are mean ± standard error of *mean* (SEM) of 20 mice (10 males and 10 females) for treated mice and 20 mice (10 males and 10 females) for non-treated mice. The data from treated mice were compared to the vehicle mice using an unpaired Student’s *t*-test. Significant differences are marked as * (*p* < 0.05), ** (*p* < 0.01).

Glucose, urea, creatinine, Sodium (Na), potassium (K), chloride (Cl), total proteins, albumin, calcium (Ca), total bilirubin, total cholesterol, triglycerides, creatinine kinase (CK), aspartate amino transferase (ASAT), alanine amino transferase (ALAT), alkaline phosphatase (ALP) and α-amylase were measured on blood collected after 4 h fasting (Figure 8). These parameters make it possible to detect potential toxic exposure of the organism [32].

Glucose levels were slightly significantly higher in male treated mice compared to the male vehicle mice. The difference is smaller in the case of female mice.

However, Loots et al. reported that the extracts of *Aloe greatheadii* reduced plasma glucose [33].

Total bilirubin levels were slightly significantly higher in female treated mice compared to the female vehicle mice. These changes could be considered physiological. CK activity was significantly higher in female treated mice compared to the vehicle mice, while no significant change was observed in males. It must be noted that there was an important variability between mice in CK levels in the same group.

The other enzymatic activities, ASAT, ALAT, ALP, and α-amylase, were not significantly changed in treated mice compared to the vehicles; however, a trend toward an increase in ASAT could be noted in female treated mice. Ca levels were significantly higher in male treated mice compared to the vehicles, while no change was observed in females.

No change was observed in total cholesterol, triglycerides, urea, creatinine, Na, K, and Cl levels between treated and vehicle mice in both sexes.

### 2.7. Blood Hematology

A complete blood cell count was performed on blood collected at the end of the study (Table 3). This analysis is a useful index to assess the toxicity of the plant extract in animals and humans [34].

Total leukocytes, erythrocytes, and platelets counts were comparable between treated and vehicle mice, and no abnormalities were observed in the morphology of the blood cells. Hemoglobin and hematocrit were similar between treated and vehicle mice. The different lineage of leukocytes: Lymphocytes, neutrophils, eosinophils, and monocytes were not significantly changed in treated mice compared to the vehicle.

### 2.8. Histology

**Figure 9:**
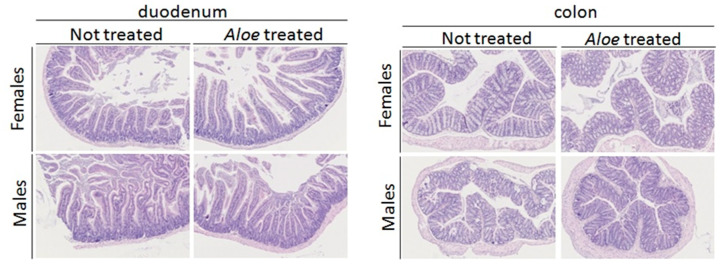
Histological structure of the duodenum and the colon cells showing any changes between treated and not treated mice with *A. djiboutiensis* latex at a concentration of 200 mg/kg.

Histopathological analysis of duodenum, ileum, and colon showed no significant microscopic change or lesion for both sex and treatments. All animals were morphologically normal for the organs considered in this study (Figure 9).

We note the absence of toxicity of ADL, the latex of *A. djiboutiensis*, at 200 mg/kg for the mice tested. To assess possible toxicities of this dose on a human scale, we used the transposition defined by the US Food and Drug Administration [35]. For a person with an average weight of 60 kg, this safety dose corresponds to 18 mg/kg. Generally, the traditional consumption of latex is below this amount. In fact, the latex used is extracted from a single leaf of a plant around three years old. Due to its bitter taste, its consumption is limited to few drops.

## 3. Materials and Methods

### 3.1. Plant Material and Extract Preparation

*Aloe djiboutiensis* (AD) leaves were collected in Goda Mountain, Northern Djibouti, at an altitude of 1500 m. The latex (ADL) was collected drop by drop after cutting the leaves, then lyophilized and stored at −5 °C before chemical and biological analysis.

Furthermore, leaves of this plant have been freeze-dried and then crushed. 100 g of *A. djiboutiensis* powder is extracted into Soxhlet apparatus for 7 h with 700 mL of methanol (ADM) and then evaporated under reduced pressure to dryness to obtain 2.56 g.

500 mg of dry latex were solubilized in the methanol, and then the sample was subjected to fractionation using 10 g of LH-20 and the methanol as mobile phase. Forty-four tubes were collected, and in total five fractions are obtained according to the chemical profile evaluated using TLC. TLC is realized using silica gel as stationary phase and a mixture of ethyl acetate, methanol, water (100:13.5: 10 *v*/*v*) as mobile phase. The five fractions were: FR-I (49.2 mg), FR-II (15.1 mg), FR-III (9.8 mg), FR-IV (36.9 mg), FR-V (27.5 mg). All the fractions are analyzed by LC-MS/MS and by NMR.

### 3.2. Chemicals

1,1-Diphenyl-2-picrylhydrazyl (DPPH), 2,20-azinobis-3-ethylbenzothiazoline-6-sulfonic acid (ABTS), Vitamin C, 6-hydroxy-2,5,7,8-tetramethylchroman-2-carboxylic acid (Trolox), iron (II) sulfate (FeSO_4_), iron (III) chloride (FeCl_3_), Folin–Ciocalteu reagent (FC reagent), quercetin, gallic acid, hydrochloric acid (HCl), potassium persulfate, phosphate buffered saline (PBS) and organics solvents, were purchased from Sigma Aldrich, Saint Quentin Fallavier, France and from Acros organics, part of Thermo Fischer, Illkirch, France.

### 3.3. Antioxidant Activity Tests

#### 3.3.1. DPPH Radical-Scavenging Test

The free-radical-scavenging activity of ADM and ADL were measured using an improved DPPH (2,2-diphenyl-1-picrylhydrazyl) assay [36], and following the protocol previously published [37]. Ascorbic acid (vitamin C) and Trolox were used as positives standards.

The IC_50_ value was calculated from the linear regression of plots of concentration of the test sample against the mean percentage of the antioxidant activity obtained from the three replicate assays. The results were expressed as mean ± SEM, and the IC_50_ values obtained from the regression plots (using Microsoft Excel) and had a good coefficient of correlation (R2 = 0.951 for ADM; R2 = 0.932 for ADL; R2 = 0.911 for vitamin C and R2 = 0.946 for Trolox).

#### 3.3.2. ABTS Radical-Scavenging Test

The ability of the samples to scavenge ABTS (2,2′-azino-bis(3-éthylbenzothiazoline-6-sulphonique) radical was determined according to previously published methods [37,38]. Ascorbic acid (vitamin C) and Trolox were used as positives standards.

The IC_50_ value was calculated from the linear regression of plots of concentration of the test sample against the mean percentage of the antioxidant activity obtained from three replicate assays. The results were expressed as mean ± SEM and the IC_50_ values obtained from the regression plots (using Microsoft Excel) and had a good coefficient of correlation (R2 = 0.934 for ADM; R2 = 0.912 for ADL; R2 = 0.801 for vitamin C and R2 = 0.939 for Trolox).

#### 3.3.3. FRAP Assay

The FRAP (ferric reducing-antioxidant power) assay was carried out according to the procedure of [39] with slight modifications [37]. The standard curve was constructed using vitamin C solution (0.065–33.3 µg/mL), and the results were expressed as µmol EVC/g dry weight of the extract. All the measurements were taken in triplicate, and the mean values were calculated.

### 3.4. Determination of Phenolic Content (PC) and Flavonoid Content (FC)

The experimental protocols of the evaluation of PC and FC were described in our previous work [40].

The results of PC were estimated using a standard curve prepared using gallic acid and expressed as milligram GAEs per gram of extract on a dry weight basis. Quercetin was used as a reference standard, and the total flavonoid content was expressed as milligrams of quercetin equivalents (mg QE/g extract).

### 3.5. LC-MS/MS Analysis, Creation of Molecular Networking and NMR Apparatus

The LC system consisted of a U3000-Dionex apparatus with an injector comprising a 1 μL loop and a UV detector at 280 nm. The LC analytical column used was an Hypersil Gold (100 mm × 2.1 mm, Thermo Scientific, Bellefonte, PA, USA) and eluted at a flow rate of 200 µL/min using a gradient 0 mn 5%B/5 mn 5%B/40 mn 99%B/45 mn 99%B/50 mn 5%B/55 mn 5%B. Solvent A consisted of water/2% of formic acid (HCOOH), and solvent B consisted of acetonitrile (ACN). The oven temperature was set at 40.00 °C, and 2 µL was injected. The LC-MS analysis was performed using a micrOTOF_Q_^TM^ apparatus (Bruker Daltonics, Bruker, Bremen, Germany), and the MS/MS data are obtained using Electrospray Ionization—High Resolution Mass Spectrometry (ESI-HRMS). A mass range of 50–1000 *m/z* and collision energy of 20 eV was used. The raw data are converted using Bruker DataAnalysis; each data is calibrated with sodium formate. All MS/MS data are converted in a mascot generic file (.mgf) file.

The analyses were performed in mass spectrometry of the L2CM laboratory at the University of Lorraine, France.

The .mgf file is sent to the GNPS website [41]. A molecular network was created using the online workflow on the GNPS platform (http://gnps.ucsd.edu). The data were filtered by removing all MS/MS fragment ions within +/− 17 Da of the precursor *m*/*z*. MS/MS spectra were window filtered by choosing only the top 6 fragment ions in the +/− 50 Da window throughout the spectrum. The precursor ion mass tolerance was set to 2.0 Da and an MS/MS fragment ion tolerance of 0.05 Da. A network was then created where edges were filtered to have a cosine score above 0.7 and more than three matched peaks. Further, edges between two nodes were kept in the network if and only if each of the nodes appeared in each other’s respective top 10 most similar nodes. Finally, the maximum size of a molecular family was set to 100, and the lowest-scoring edges were removed from molecular families until the molecular family size was below this threshold. The spectra in the network were then searched against GNPS’ spectral libraries. The library spectra were filtered in the same manner as the input data. All matches kept between network spectra, and library spectra were required to have a score above 0.7 and at least three matched peaks. In addition, DEREPLICATOR+ (PLUS) [41], a bioinformatic tool available on GNPS, is used to allow the annotation of non-peptidic natural products in MS/MS data using in silico fragmentation tree. For the visualization of molecular networking, the software Cytoscape^®^ (version 3.8.2) is used.

^1^H and ^13^C-NMR spectra were recorded on a Bruker Avance III 400 spectrometer (Bruker BioSpin, Rheinstetten, Germany), operating at a frequency of 400.13 MHz at a temperature of 26 °C using a BBFO Probe and a Bruker sample changer. The samples are dissolved in MeOD solvent. NMR analyses were performed on the «Plateforme de RMN de l’Institut Jean Barriol», University of Lorraine, France.

### 3.6. In-Vitro Toxicity

The toxicity of two samples (ADM and ADL) were evaluated on MRC-5 cells, a fibroblast-derived from normal lung tissue (MRC-5 pd30 ECACC 05090501), using the MTT test [42], based on the reduction of MTT by succinate dehydrogenase in formazan crystals in living cells. The protocol used is adapted from [43].

### 3.7. In Vivo Toxicity

#### 3.7.1. Animal and Ethics

This study follows French and European Legislation (European Directive 2010/063 EU, French «Décret n° 2013-118» about the protection of animals used for scientific purposes). All animal experiments were conducted in accordance with ethical standards and were approved by the Ethics Committee of research French minister under reference APAFIS#15626-2018062118083591 v4.

#### 3.7.2. Study Design

The study was performed on 40 CD1 mice (20 males and 20 females), derived from Lynch’s Swiss mice, by Roswell Park Memorial Institute, Buffalo, New York [44]. Mice arrived at the age of 10 weeks, were housed 3 to 4 per cage, and fed with a standard chow diet (D04, Safe). Six weeks later, half of the mice (10 males and 10 females) were treated by oral gavage (10 mL/kg) with a suspension of *A. djiboutiensis* latex at a concentration of 200 mg/kg in phosphate buffered saline (PBS). PBS (10 mL/kg) was administered by oral gavage to the control vehicle mice (10 males and 10 females). One week after treatment, mice were submitted to a dysmorphological screen; this test is performed to examine morphological abnormalities with respect to general physical appearance and body shape on mice. At the same time, rectal body temperature was measured. The same day mice were fasted for 4 h (from 7:00 am to am 11:00), and blood was collected by a retro orbital puncture for evaluation of blood chemistry and hematology. In the end, the mice were sacrificed. The in vivo toxicity was performed on CD1 mice by oral gavage with a suspension of *A. djiboutiensis* latex at a concentration of 200 mg/kg in phosphate buffered saline (PBS).

#### 3.7.3. Dysmorphological Screen

The test is performed to examine morphological abnormalities with respect to general physical appearance and body shape on mice: Weight, length, and obvious dysmorphologies in the physical appearance, i.e., tail kinks, shape of ears, eyes, head, teethes, limbs, number and shape of digit, irregularities and variation in coat color, hair distribution and development, irregularities in the genitals [45]. The test was conducted during the light period in vigil-fed mice.

#### 3.7.4. Body Temperature

Body temperature is recorded by rectal measurement using a rectal probe.

#### 3.7.5. Blood Analysis

Blood was collected by retro orbital puncture under isoflurane anesthesia at 11h00 am on mice fasted for 4 h. The blood chemistry was performed on an OLYMPUS AU-480 automated laboratory work station (Beckmann Coulter, Brea, CA, USA) with kits and controls supplied by Beckmann Coulter. Moreover, a complete blood count was performed on total blood on an Advia 120 Vet (Siemens).

#### 3.7.6. Histology

After sacrifice, duodenum, ileum, and colon have been collected, formalin-fixed, paraffin-embedded, and 5 µm thick hematoxylin and eosin stained sections were preceded for routine histological analysis.

#### 3.7.7. Statistical Analysis

The measurements are repeated three times, and the data are expressed mean ± standard error of the *mean* (SEM). The data from treated mice were compared to the vehicle mice using an unpaired Student’s *t*-test and * *p* < 0.05), ** *p* < 0.01), *** *p* < 0.001 were accepted as a significant difference.

## 4. Conclusions

*A. djiboutiensis* is a medicinal plant recently named botanically. Despite the strong use of this species in traditional medicine in Djibouti, there are no data on its possible toxicity, biological effects, or chemical constituents.

For the first time, we report the antioxidant activity, phytochemical investigation, biological activity, and toxicity of the latex and methanol leaves extract of *A. djiboutiensis*.

Surprisingly, a weak antioxidant activity was measured in both extracts, while the content of phenolic compounds was high mainly in the latex. However, the phytochemical analysis showed a high level of methylation of chromones, and it is known that the antioxidant activity is different depending on these specialized metabolites.

In this study, the chemical composition of the latex of this plant was examined by molecular networking. This work enabled the detection of aloin A/B, a compound present also in *A. ferox* and *A. barbadensis*, and responsible for the laxative effect of aloe latex. Moreover, 6-malonyl nataloin, a rare anthrone nataloin, was detected in *A. djiboutiensis* latex. The identification of anthraquinones in the latex of *A. djiboutiensis* justifies its utilization in medicinal use in Djibouti as a laxative. In vivo animal experimentation consisting of the treatment of mice with *A. djiboutiensis* latex at a concentration of 200 mg/kg did not show any toxicity. This treatment did not lead to any change in bodyweight and body temperature and had no impact on the morphological examination of the mice. Blood hematological parameters were not affected by the treatment; no anemia and no inflammation were detected. It appeared that the treatment had no major impact on most of the blood chemistry parameters measured (lipids, proteins, urea, creatinine, and electrolytes). *A. djiboutiensis* treatment does not induce any evident alteration of duodenum, ileum, and colon morphology in CD1 mouse. According to these results and following equations for dose conversion between animals and human [35], the traditional use of the latex of *A. djiboutiensis* is safe until at less 18 mg/kg for human level and has beneficial effects for human health. In contrast to other species of aloe, weak antioxidant activity, while the high content of phenolic compounds, observed in latex and leave extract of this plant should be explained by other phytochemical studies, such as the extraction and quantification of chromones.

## Figures and Tables

**Table 1:** Antioxidant activity, phenolic and flavonoid contents of *A. djiboutiensis* latex (ADL) and leave methanolic extract (ADM).

Antioxidant Activity				Phenolic Content (PC)	Flavonoid Content (FC)
Sample	DPPH; ABTS IC_50_ (µg/mL)		FRAP (mg EVC/g DW)	mg GAE/g DW	mg QE/g DW
ADL	>1000	600 ± 1.8	1.50 ± 0.12	83.01 ± 0.8	13.12 ± 0.11
ADM	>1000	632 ± 3.2	1.68 ± 0.20	46.4 ± 0.32	4.25 ± 0.1
Vitamin C	220 ± 3.1	50 ± 3.3	-	-	-
Trolox	130 ± 7.1	50 ± 2.7	-	-	-

Phenolic and flavonoid are exprimed as GAE—gallic acid equivalent and QE—quercetin equivalent, respectively. Vitamin C was used as a reference to the FRAP test, equivalent vitamin C (EVC). Values are representative of three independent determinations. *p* values ≤ 0.05.

**Table 2:** Putative identification of chemical compounds in latex and selected fractions of the latex of *A. djibutiensis* by LC-M/MS.

Compound Label	Name	Retention Time in Minutes	Chemical Formula [M + H]^+^	Mesured *m/z* (Da) [M + H]^+^	Error (ppm)	MS/MS Fragmentation	Cluster Number	Presence in the Samples
**Anthraquinones Derivatives**								
Cmpds **1A** and **1B** ^DT^	Aloin A/B	14.7/15.1	[C_21_H_20_O_9_ + H]^+^	419.1441	0.4	257.0796 [M-glycosyl + H]^+^ 239.0768 [M-glycosyl-H_2_O + H]^+^ 211.0785 [M-glycosyl-H_2_O-CO + H]^+^	A	ADL, FR-II FR-III, FR-IV, FR-V
Cmpds **2A** and **2B ^D+^**	Aloinoside A/B	15.0/15.7	[C_27_H_32_O_13_ + H]^+^	565.1915	6.4	419.1416 [M-rhamnosyl + H]^+^ 239.0730 [M-rhamnosyl-glycosyl + H]^+^ 211.0737 [M-rhamnosyl-glycosyl-CO + H]^+^	B	ADL FR-II, FR-III
Cmpd **3 ^M^**	Malonyl nataloin	15.6	[C_24_H_24_O_12_ + H]	505.1374	6.6	239.0697 [M-mal-glycosyl + H]^+^	C	ADL FR-IV, FR-V
Cmpd **4**	Aloin-pentose	16.9	[C_20_H_20_O_8_ + H]^+^	389.1213	4.6	239.0676 [M-pentosyl-H_2_O + H]^+^	C	ADL FR-II, FR-III FR-IV, FR-V
Cmpd **5**	Endocrocin	13.4	[C_16_H_10_O_7_ + H]^+^	315.0478	6.9	297.0369 [M-H_2_O + H]^+^ 269.0442 [M-H_2_O-CO_2_ + H]^+^ 241.048 [M-H_2_O-2CO_2_ + H]^+^	D	ADL FR-I, FR-II, FR-III, FR-IV, FR-V
Cmpd **6 ^DT^**	Aloemodin	19.1	[C_15_H_10_O_5_ + H]^+^	271.0582	7.0	253.0477 [M-H_2_O + H]^+^ 241.0465 [M-CH_2_O + H]^+^ 225.0526 [M-H_2_O-CO + H]^+^	D	ADL FR-I, FR-II, FR-III, FR-IV, FR-V
Cmpd **7**	Citreorosein	17.4	[C_15_H_10_O_6_ + H]^+^	287.0531	6.7	269.0454 [M-H_2_O + H]^+^ 241.0487 [M-H_2_O-CO + H]^+^ 213.0537 [M-H_2_O-2CO + H]^+^ 185.0578 [M-H_2_O-3CO + H]^+^ 157.0654 [M-H_2_O-4CO + H]^+^	D	ADL FR-IV, FR-V
**Chromones Derivatives**								
Cmpd **8 ^D+^**	Isoaloeresin D or Aloesinol_7-Me_ether,_2″-*O*-(4-hydroxy-*E*-cinnamoyl)	15.4	[C_29_H_32_O_11_ + H]^+^	557.1998	9.5	513.1783 [M-CH_2_CHOH + H]^+^ 349.1291 [M-CH_2_CHOH-coumaric + H]^+^ 217.0876 [M-CH_2_CHOH-coumaric-pentosyl + H]^+^ 187.0727 [M-CH_2_CHOH-coumaric-glycosyl + H]^+^ 437.1589 [M-part of sugar + H]^+^ 393.1374 [M-part of sugar-CH_2_CO + H]^+^ 247.0963 [M-part of sugar-CH_2_CO-(coumaric-H_2_O) + H]^+^ 147.0485 [coumaric-H_2_O + H]^+^	E	ADL FR-I, FR-II; FR-III, FR-IV, FR-V
Cmpds **9A** and **9B** ^D+^	Aloesinol_2″-*O*-(4-Methoxy-cinnamoyl)	16.0/17.0	[C_29_H_32_O_11_ + H]^+^	557.2014	0.7	513.1783 [M-CH_2_CHOH + H]^+^ 335.1101 [M-CH_2_CHOH-MeO-cinnamoyl + H]^+^ 203.0672 [M-CH_2_CHOH-MeO-cinnamoyl-pentosyl + H]^+^ 161.059 (MeO-cinnamoyl)^+^	F	FR-I
Cmpds **10A** and **10B ^D+^**	Isoloeresin-D + glycosyl	12.6/13.2	[C_35_H_42_O_16_ + H]^+^	719.2570	4.5	557.2033 [M-glycosyl + H]^+^ 393.1326 [M-2 glycosyl + H]^+^ 247.0962 [M-2 glycosyl-(coumaric-H_2_O) + H]^+^ 147.0427 [Coumaric acid-H_2_O + H]^+^	G	ADL FR-I
Cmpd **11**	Aloesol	13.5	[C_13_H_14_O_4_ + H]^+^	235.0951	6.9	191.0684 [M-C_2_H_4_O + H]^+^ 176.0466 [M-C_2_H_4_O-CH_3_ + H]^+^	No cluster	FR-III
Cmpd **12**	Aloesin	12.4	[C_19_H_22_O_9_ + H]^+^	395.1339	0.6	233.0786 [M-glycosyl + H]^+^ 215.068 [M-glycosyl-H_2_O + H]^+^ 203.0666 [M-glycosyl-CH_2_O + H]^+^	H	ADL
Cmpd **13**	Aloesin–Glycoside	9.6	[C_25_H_32_O_14_ + H]^+^	557.1874	1.6	395.1321 [M-glycosyl + H]^+^ 233.0780 [M-2 glycosyl + H]^+^	H	ADL FR-I
Cmpd **14 ^D+^**	Aloesinol_7-Me_ether,_2″-*O*-(3,4-dihydroxy-*E*-cinnamoyl)	13.8	[C_29_H_32_O_12_ + H]^+^	573.1982	2.8	529.1748 [M-CH_2_CHOH + H]^+^ 367.1345 [M-CH_2_CHOH-(caffeic-H_2_O) + H]^+^ 205.0817 [M-CH_2_CHOH-(caffeic-H_2_O) + H]^+^ 409.1270 [M-deoxyhexose + H]^+^ 247.0948 [M-deoxyhexose-(caffeic-H_2_O) + H]^+^ 163.0378 [caffeic-H_2_O + H]^+^	E	ADL FR-I, FR-II, FR-III, FR-IV, FR-V
Cmpd **15 ^D+^**	Aloesinol_2″-*O*-(4-Hydroxy-*E*-cinnamoyl)	13.4	[C_28_H_30_O_11_ + H]^+^	543.1908	8.6	499.1630 [M-CH_2_CHOH + H]^+^ 335.1095 [M-CH_2_CHOH-coumaric + H]^+^ 203.0659 [M-CH_2_CHOH-coumaric-pentosyl + H]^+^ 397.1467 [M-(coumaric-H_2_O) + H]^+^ 233.0781 [M-(coumaric-H_2_O)-deoxyhexose + H]^+^ 379.1258 [M-deoxyhexose + H]^+^ 147.0485 [coumaric-H_2_O + H]^+^	I	ADL FR-I, FR-II, FR-III
**Flavonols/Chromones Isomers**								
Cmpd **16**	Eupatorin or aglycon of pendulin	16.4	[C_18_H_16_O_7_ + H]^+^	345.0953	4.5	285.0768 [M-H_2_O-CH_2_CO + H]^+^ 267.0615 [M-H_2_O-CH_2_CO-H_2_O + H]^+^	L	ADL FR-IV, FR-V
Cmpd **17**	Eupatorin-glycosyde or Pendulin	13.6	[C_24_H_26_O_12_ + H]^+^	507.1505	1.5	345.0959 [M-glycosyl + H]^+^ 327.0858 [M-glycosyl-H_2_O + H]^+^ 285.0768 [M-glycosyl-H_2_O-CH_2_CO + H]^+^	M	ADL FR-I, FR-II, FR-III, FR-IV, FR-V

**Table 3:** Biochemical changes in blood hematology, complete blood cell count in mice given latex of *A. djiboutiensis* (ADL).

Parameters		Dosage (g/kg/day)			
		Male		Female	
	Unit	0	0.15	0	0.15
WBC	×10^3^ cells/µL	5.60	7.07	6.54	5.79
RBC	×10^6^ cells/µL	8.65	9.01	9.35	9.56
HGB	g/dL	12.8	13.4	14.4	14.1
HCT	%	45.6	47.6	49.8	49.0
MCV **	fL	52.8	52.9	53.3	51.3
MCH *	pg	14.8	14.9	15.4	14.8
MCHC	g/dL	28.0	28.2	28.9	28.9
NEUTRO	%	17.6	19.8	13.4	18.8
LYMPHO	%	76.2	74.0	80.0	74.6
MONO	%	3.1	2.1	1.5	1.9
EOSINO	%	2.6	3.6	4.7	4.5
LUC	%	0.4	0.4	0.3	0.1
BASO	%	0.1	0.1	0.1	0.1
PLT	×10^3^ cells/µL	1232	1307	1090	1236
MPV	fL	4.2	4.3	4.5	4.4

(WBC) white blood cells, (RBC) red blood cells, (HGB) hemoglobin, (HCT) hematocrit, (MCV) mean corpuscular volume, (MCHC) Mean corpuscular hemoglobin concentration, (NEUTRO) granulocytes neutrophil, (LYMPHO) lymphocytes, (MONO) monocytes, (EOSINO) granulocytes eosinophil, (LUC) large unstained cells, (BASO) granulocytes basophil, (PLT) platelets and (MPV) Mean platelet volume. Data are mean ± standard error of *mean* (SEM) of 20 mice (10 males and 10 females) for treated mice and 20 mice (10 males and 10 females) for non-treated mice. The data from treated mice were compared to the vehicle mice using an unpaired Student’s *t*-test. Significant differences are marked as * (*p* < 0.05), ** (*p* < 0.01).

## Data Availability

The data presented in this study are available in supplementary material.

## Supplementary Materials

The following are available online, Figure S1: MS/MS spectrum of compound **1**, Aloin A/B; Figure S2: ^1^H-NMR spectrum of the fraction FR-II in MeOD (400 MHz); Figure S3: ^13^C-NMR spectrum of the fraction FR-II in MeOD (100 MHz); Figure S4: MS/MS spectrum of compound **8**, Isoaloeresin D; Figure S5: ^1^H-NMR spectrum of the fraction FR-I in MeOD (400 MHz); Figure S6: ^13^C-NMR spectrum of the fraction FR-I in MeOD (100 MHz); Figure S7: MS/MS spectrum of compounds **9A**/**9B**, Aloesinol_2″-*O*-(4-Methoxy-cinnamoyl) derivatives; Figure S8: MS/MS spectrum of compounds **10A** and **10B**, Isoaloeresin D glucosyle derivatives.

## Sample Availability

Samples of the compounds are not available from the authors.
