# Supplementary material for: Aloe djiboutiensis: Antioxidant Activity, Molecular Networking-Based Approach and In Vivo Toxicity of This Endemic Species in Djibouti
Source: Molecules. 2021 May 20;26(10):3046. doi: 10.3390/molecules26103046 (PMC8161010; doi:10.3390/molecules26103046)

Supplementary materials

Aloe djiboutiensis: antioxidant activity, molecular networking-based dereplication and *in vivo* toxicity of this endemic species in Djibouti

Abdirahman Elmi[1,2], Fatouma M. Abdoul-Latif[2], Rosella Spina[1], Francois Dupire[1], Stephanie Philippot[1], Marie-France Champy[3], Hugues Jacobs[3], Dominique Laurain-Mattar[1]*

1 Université de Lorraine, CNRS, L2CM, F-54000 Nancy, France; abelfourreh@hotmail.com (A.E.); rosella.spina@univ-lorraine.fr (R.S.); francois.dupire@univ-lorraine.fr (F.D.); stephanie.philippot@univ-lorraine.fr (S.P.);

2 Medicinal Research Institute, Centre d'Etudes et de Recherche de Djibouti, IRM-CERD, Route de l'Aéroport,Haramous B.P. 486, Djibouti; fatouma_abdoulatif@yahoo.fr (F.M.A.-l.)

3 PHENOMIN-ICS, Institut Clinique de la souris, CNRS, UMR7104, Illkirch, France ; INSERM, U1258, Illkirch, France; Université de Strasbourg, France ; champy@igbmc.fr (M.F.C.) ; hugues@igbmc.fr (H.J.).

* Correspondence: dominique.mattar@univ-lorraine.fr (D.L.M.); Tel.: +33 3 72 74 56 75

Contents

Figure S1: MS/MS spectrum of compound 1, Aloin A/B.
Figure S2: ^{1}H-NMR spectrum of the fraction FR-II in MeOD (400 MHz).
Figure S3: ^{13}C-NMR spectrum of the fraction FR-II in MeOD (100 MHz).
Figure S4: MS/MS spectrum of compound 8, Isoaloeresin D.
Figure S5: ^{1}H-NMR spectrum of the fraction FR-I in MeOD (400 MHz).
Figure S6: ^{13}C-NMR spectrum of the fraction FR-I in MeOD (100 MHz).
Figure S7: MS/MS spectrum of compounds 9 A/B, Aloesinol_2''-O-(4-Methoxy-cinnamoyl) derivatives.
Figure S8: MS/MS spectrum of compounds 10 A/B, Isoaloeresin D glucosyle derivatives.

Figure S1: MS/MS spectrum of compound 1, Aloin A/B.

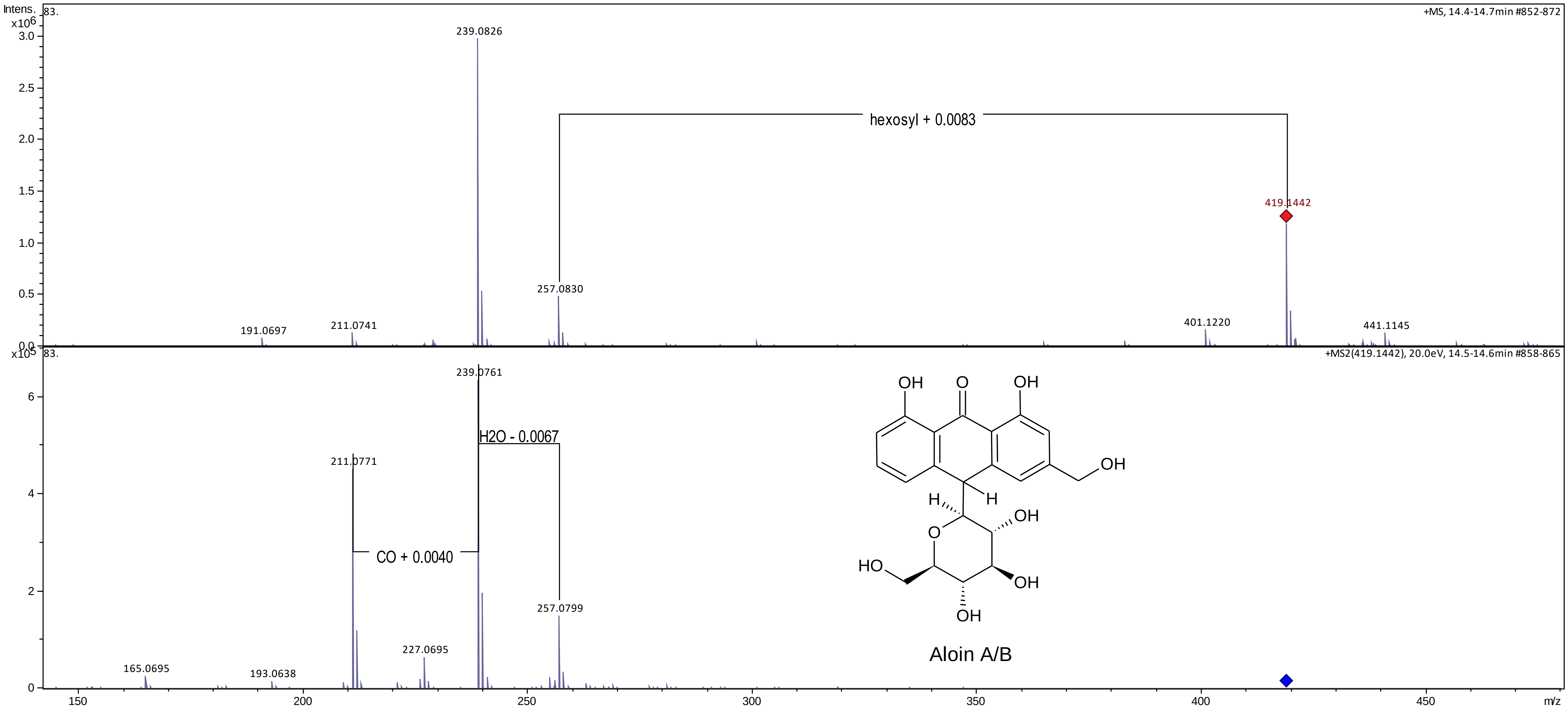

Figure S2: ^{1}H-NMR spectrum of the fraction FR-II in MeOD (400 MHz).
The characteristic signals of compound 1, Aloin A/B, are visible.

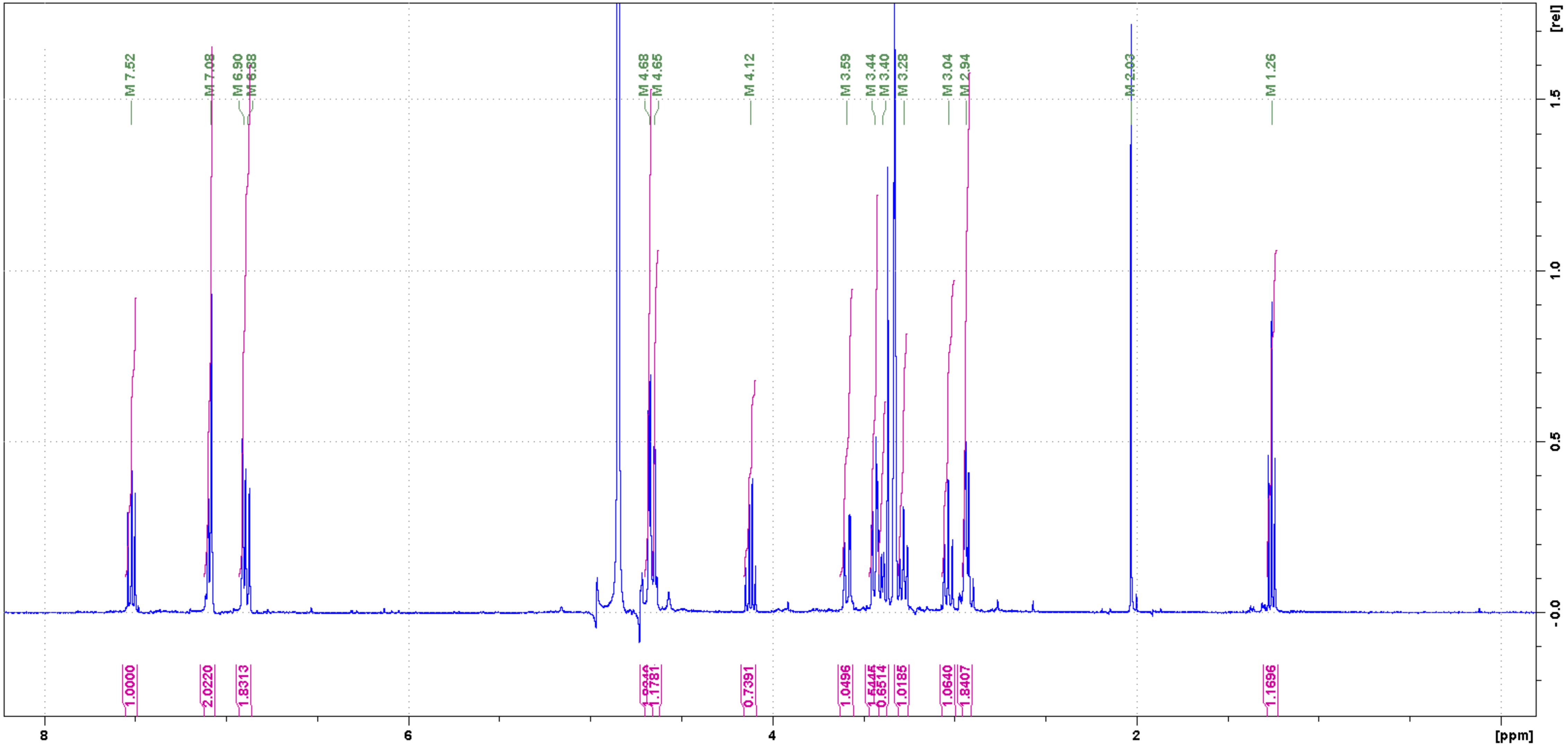

Figure S3: ^{13}C-NMR spectrum of the fraction FR-II in MeOD (100 MHz).
The characteristic signals of compound 1, Aloin A/B, are visible.

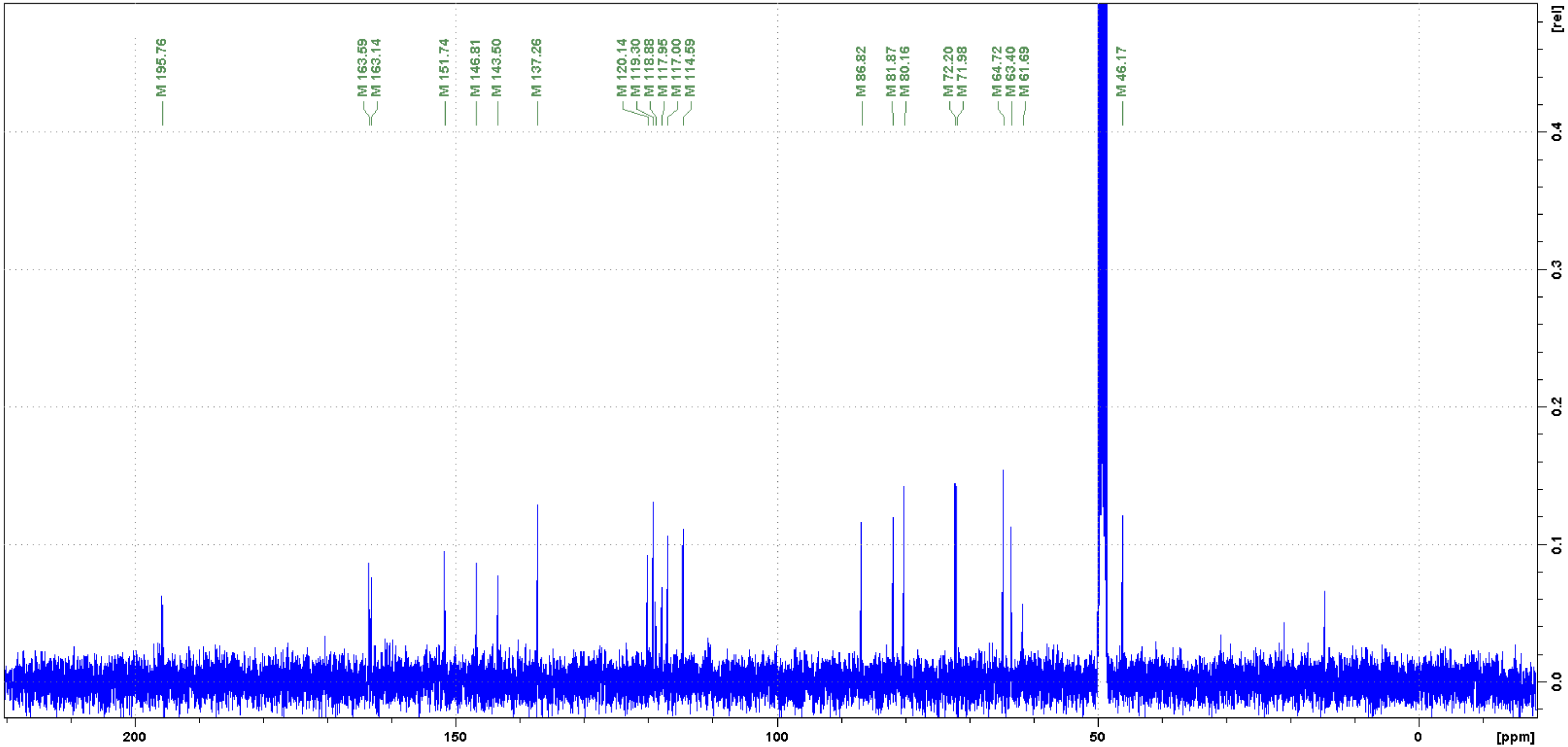

^{13}C NMR (100 MHz, CD_3OD): δ (ppm) 195.7, 163.6, 163.1, 151.7, 151.7, 146.8, 143.5, 137.3, 120.1, 119.3, 118.9, 117.9, 117.0, 114.6, 86.8, 81.9, 80.2, 72.2, 72.0, 64.7, 63.4.

Figure S4: MS/MS spectrum of compound 8, Isoaloeresin D.

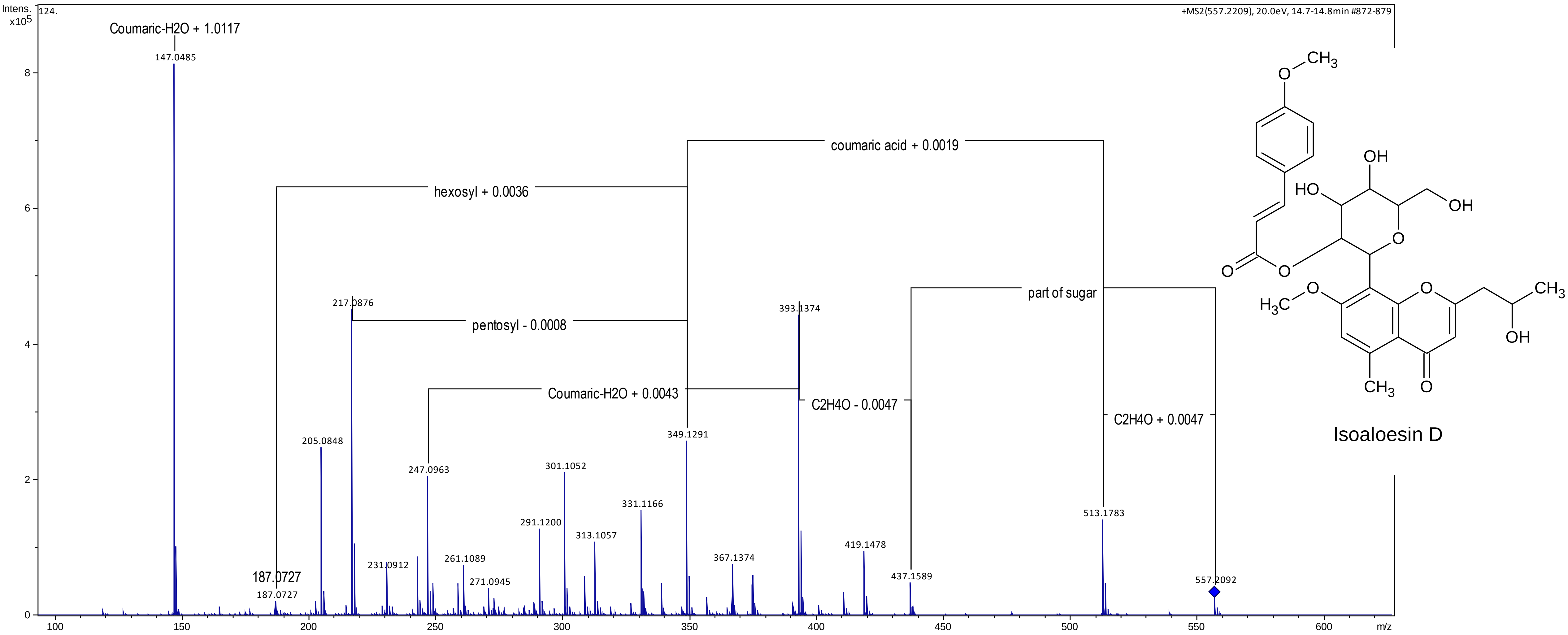

Figure S5: ^{1}H-NMR spectrum of the fraction FR-I in MeOD (400 MHz).
The characteristic signals of the compound 8, Isoaloeresin D, are visible.

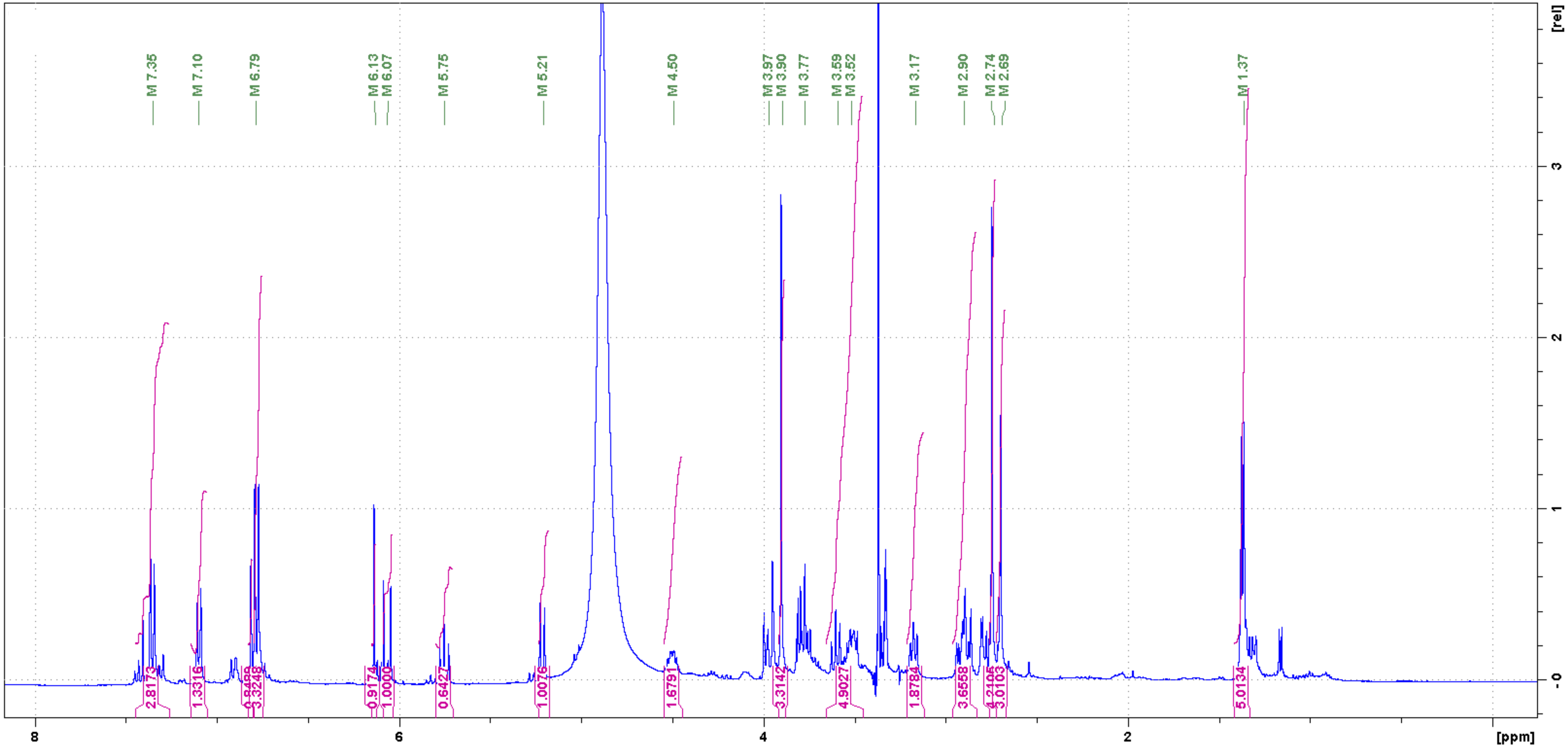

Figure S6: ^{13}C-NMR spectrum of the fraction FR-I in MeOD (100 MHz).
The characteristic signals of the compound 8, Isoaloeresin D, are visible.

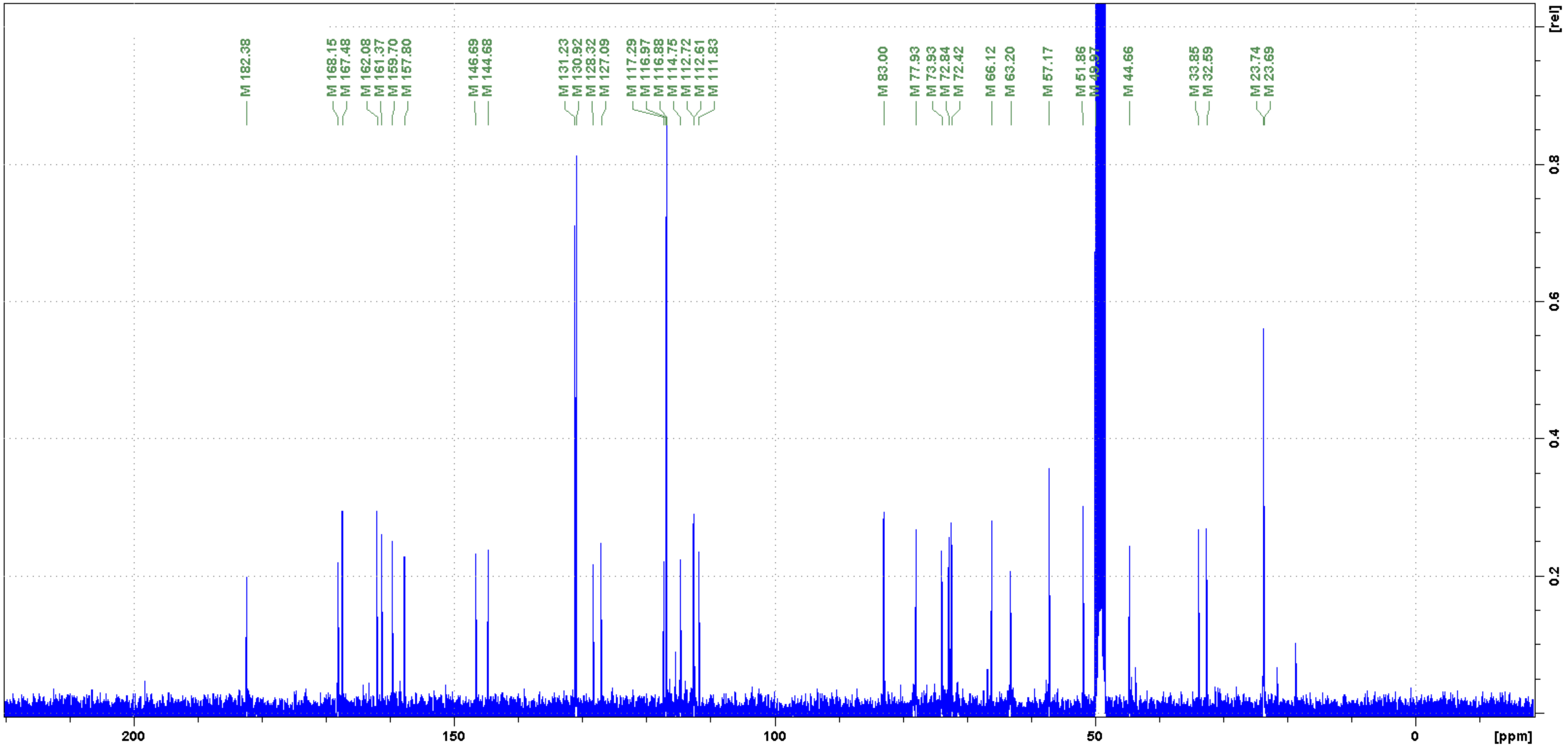

^{13}C NMR (100 MHz, CD_3OD): δ (ppm) 182.4, 168.1, 167.5, 162.1, 161.4, 159.7, 146.7, 144.7, 131.2, 130.9, 127.1, 117.3, 117.0, 116.9, 114.7, 112.7, 112.6, 111.8, 83.0, 77.9, 73.9, 72.8, 72.4, 66.1, 63.2, 57.2, 44.7, 23.7, 23.6

Figure S7: MS/MS spectrum of compounds 9 A/B, Aloesinol_2''-O-(4-Methoxy-cinnamoyl) derivatives

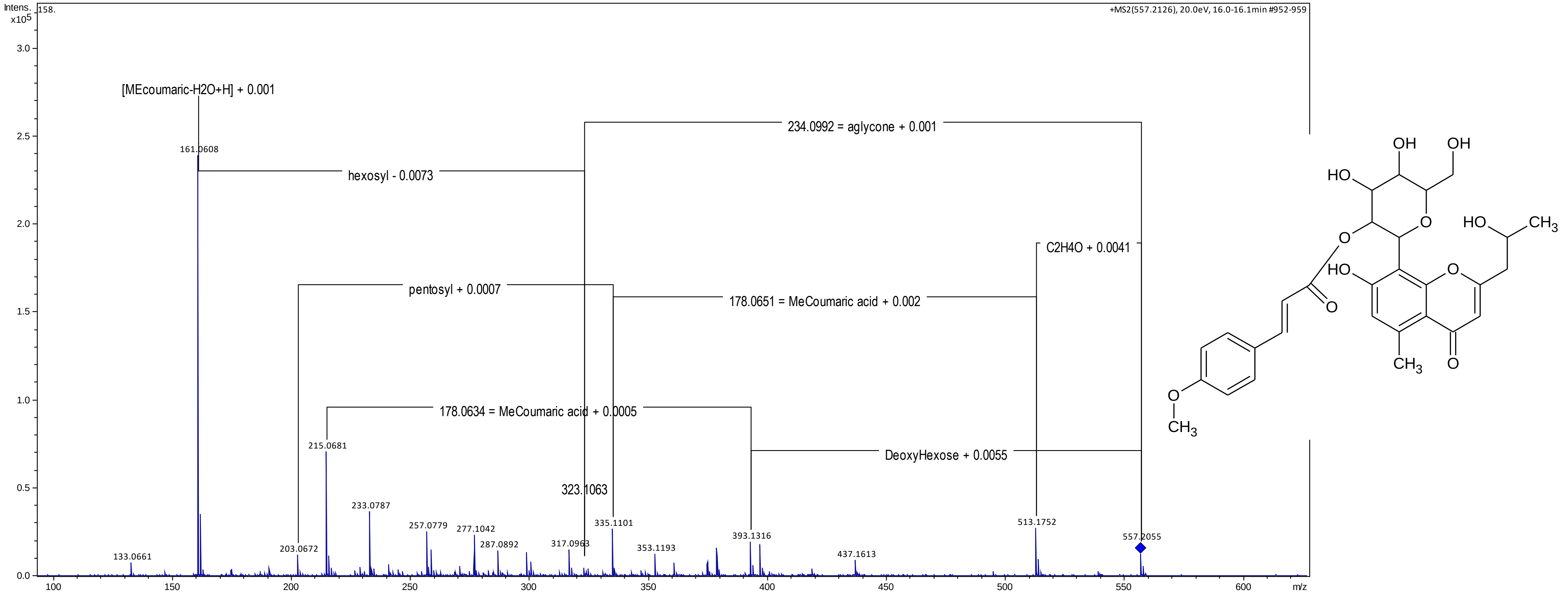

Figure S8: MS/MS spectrum of compounds 10 A/B, Isoaloeresin D glycosyle derivatives

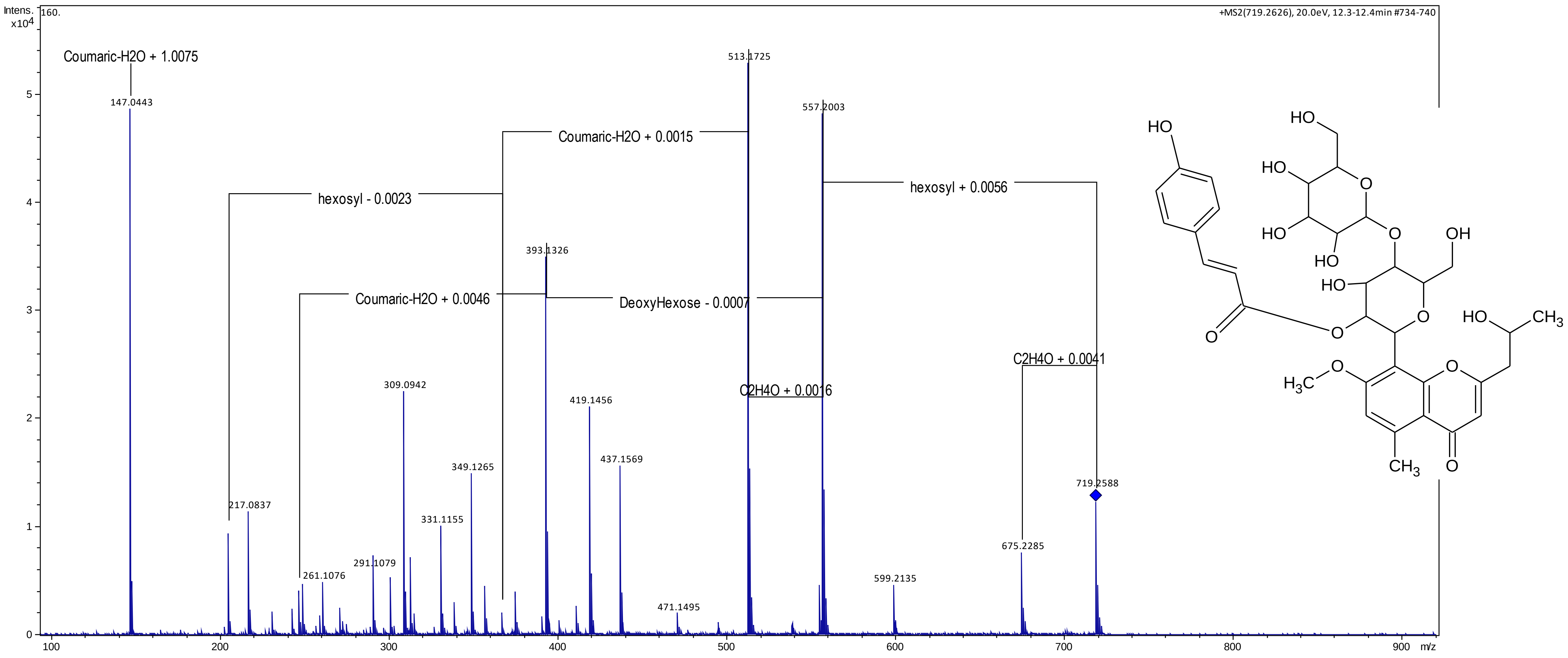

Supplement: Supplementary file 1 [file molecules-26-03046-s001.zip › molecules-1208611-supplementary.pdf]
